# Supplementary material for: Taking account of uncertainties in digital land suitability assessment
Source: PeerJ. 2015 Oct 27;3:e1366. doi: 10.7717/peerj.1366 (PMC4627905; doi:10.7717/peerj.1366)

Land suitability assessment inputs for Hazelnuts: Meander Valley, Tasmania

SOIL DEPTH

1. Probability of soil depth <1.5m and >1.5m most likely outcome.

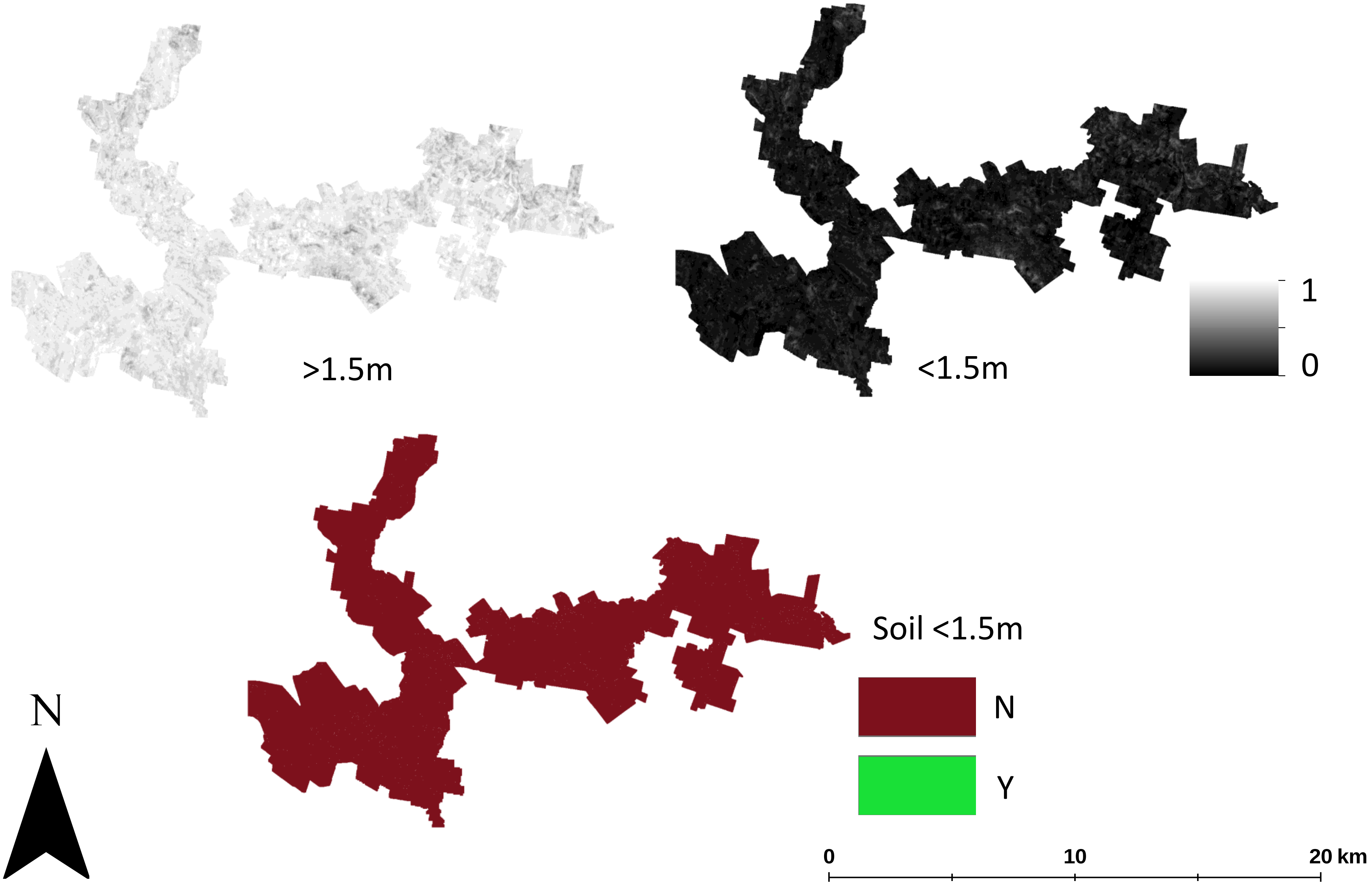

Land suitability assessment inputs for Hazelnuts: Meander Valley, Tasmania

SOIL DEPTH and 90% upper and lower prediction limits

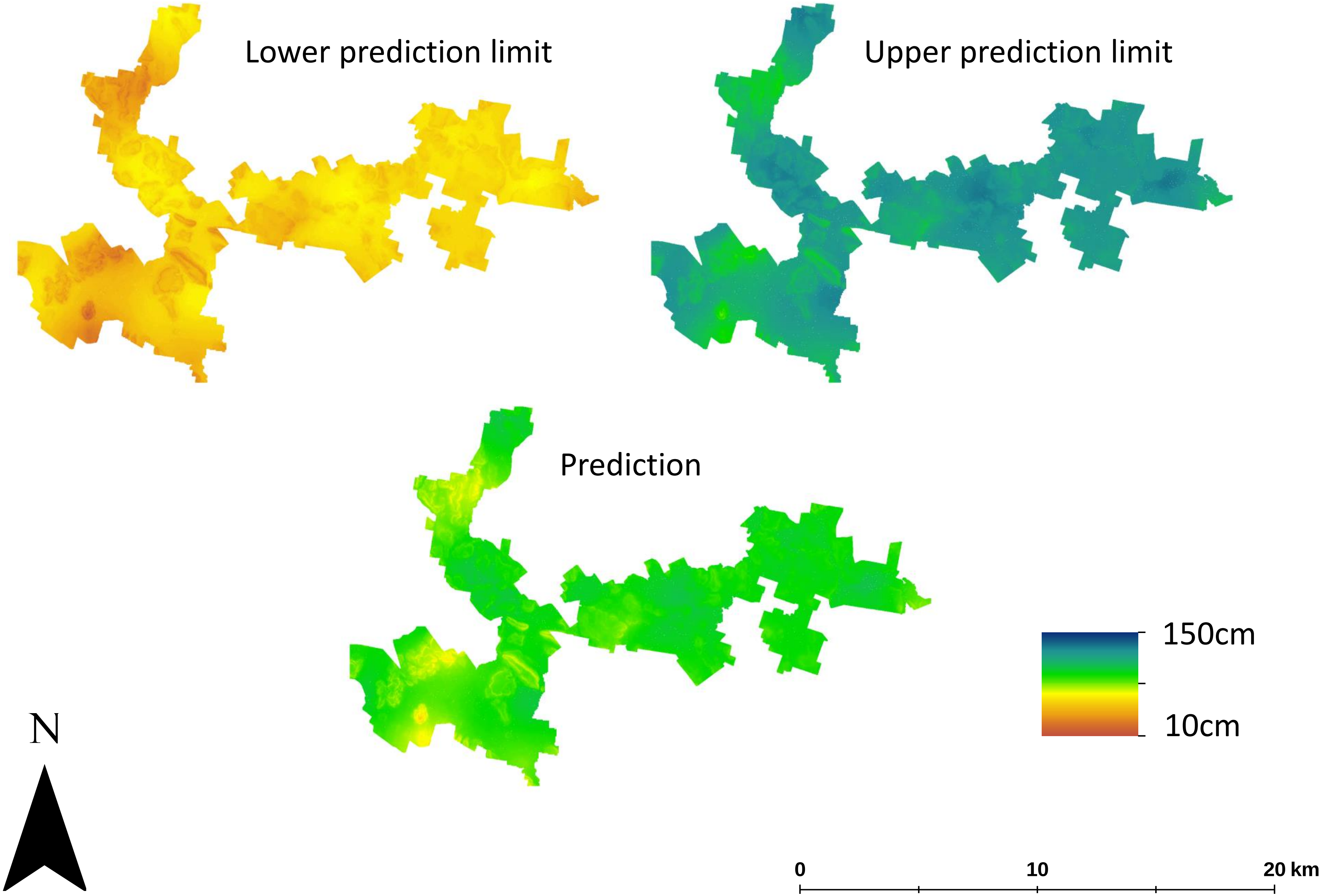

Land suitability assessment inputs for Hazelnuts: Meander Valley, Tasmania

SOIL pH (1:5 soil:water) top 15cm

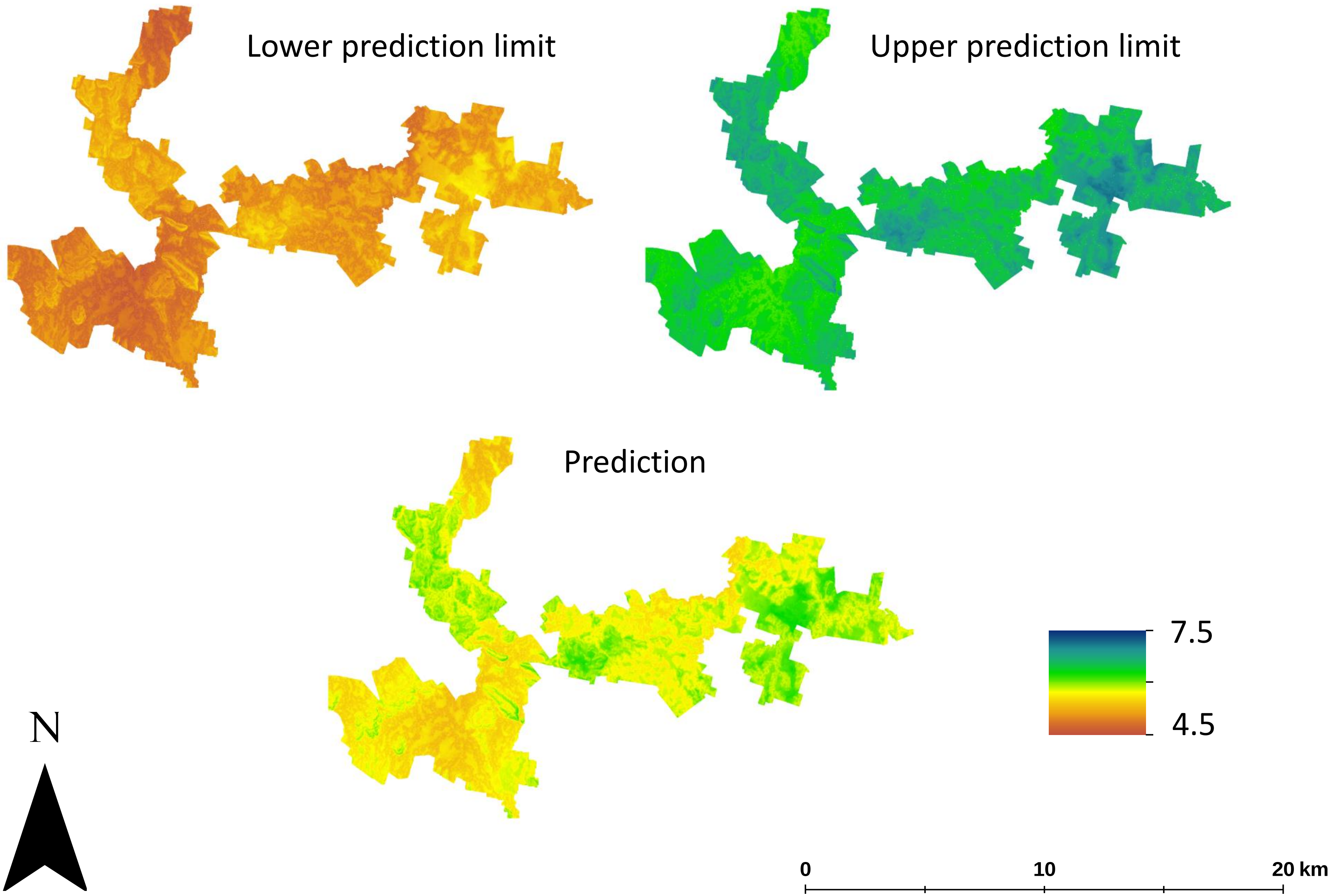

Land suitability assessment inputs for Hazelnuts: Meander Valley, Tasmania

SOIL EC (dS/m) top 15cm

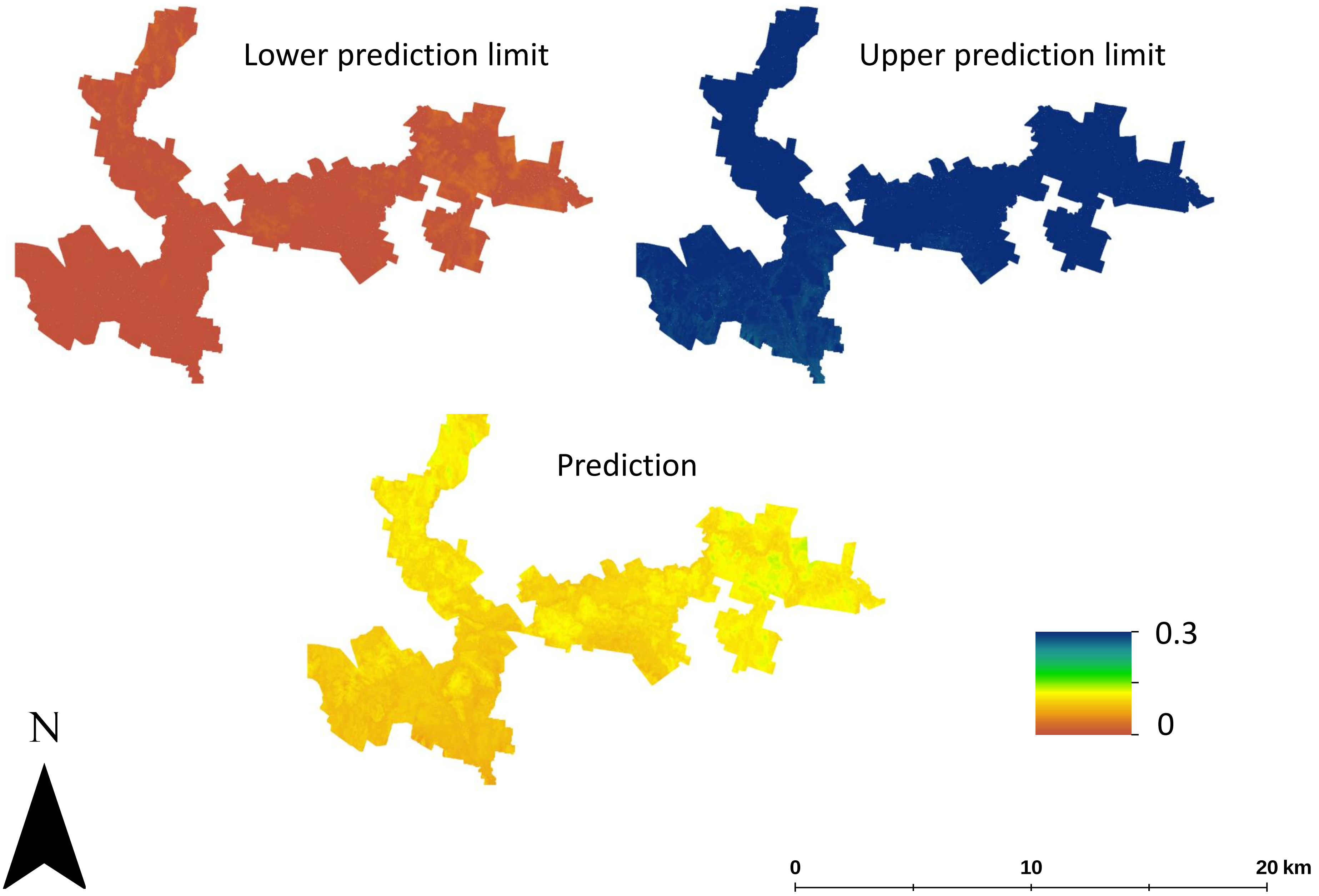

Land suitability assessment inputs for Hazelnuts: Meander Valley, Tasmania

SOIL Texture (clay %) top 15cm

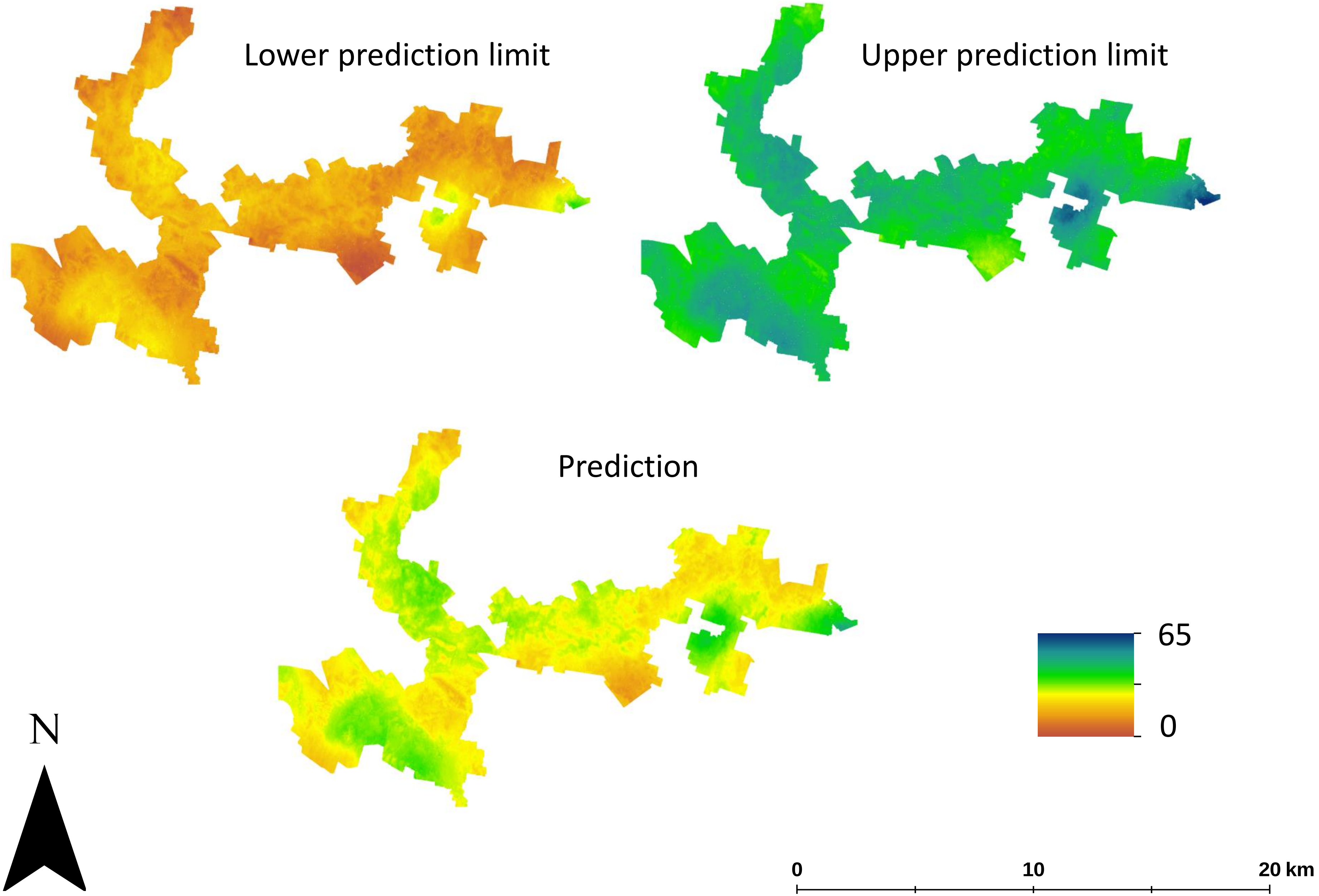

Land suitability assessment inputs for Hazelnuts: Meander Valley, Tasmania

SOIL Drainage (continuous index) 1 = Very poorly drained, 6 = Rapidly drained

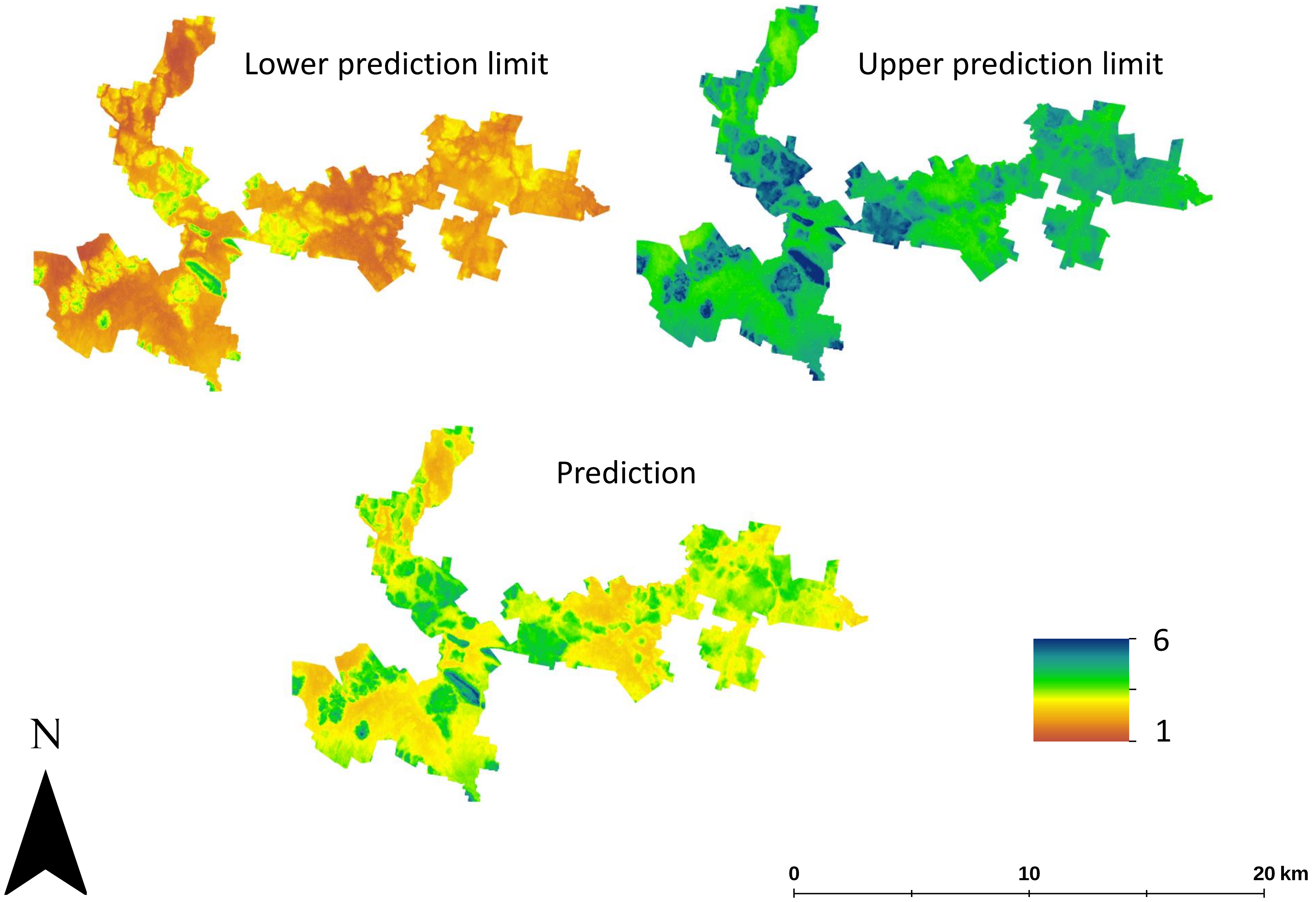

Land suitability assessment inputs for Hazelnuts: Meander Valley, Tasmania

SOIL Stoniness: Presence or absence of rock fragments to 15cm (probability and most likely outcome

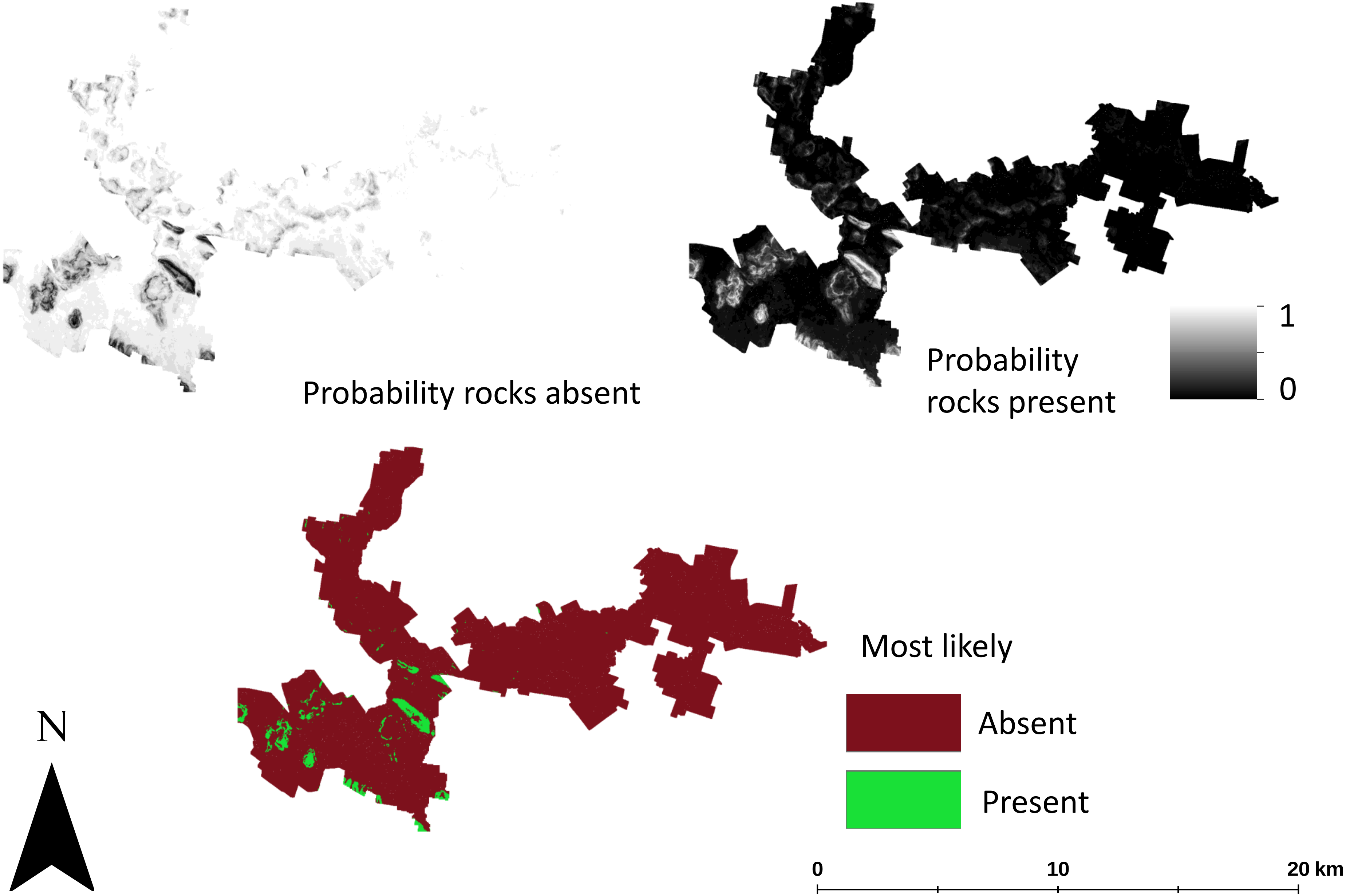

Land suitability assessment inputs for Hazelnuts: Meander Valley, Tasmania

SOIL Stoniness: Ordinal stoniness class (most likely) with probabilities

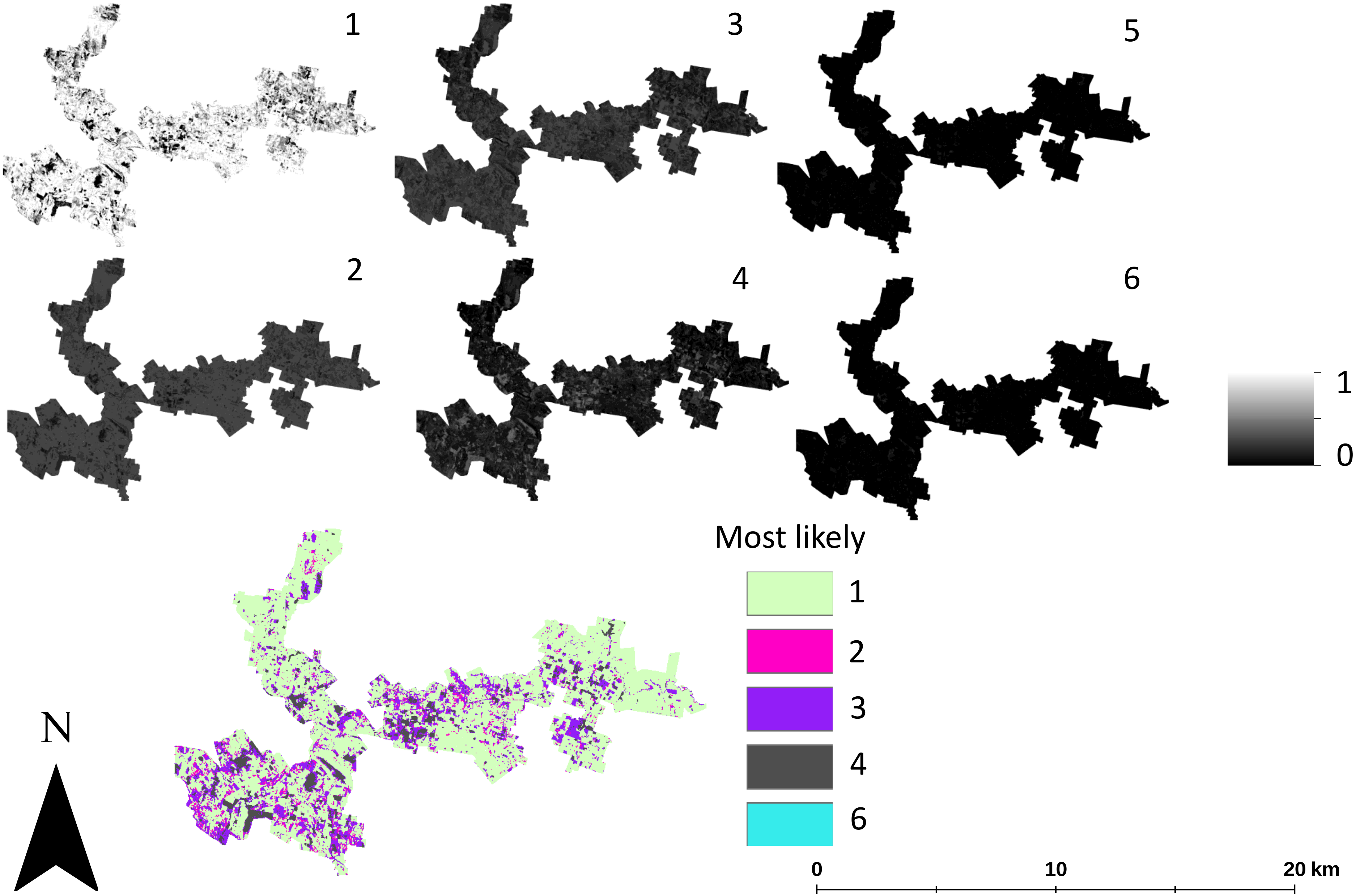

Land suitability assessment inputs for Hazelnuts: Meander Valley, Tasmania

Incidence of frost occurrence. Likely or unlikely to occur

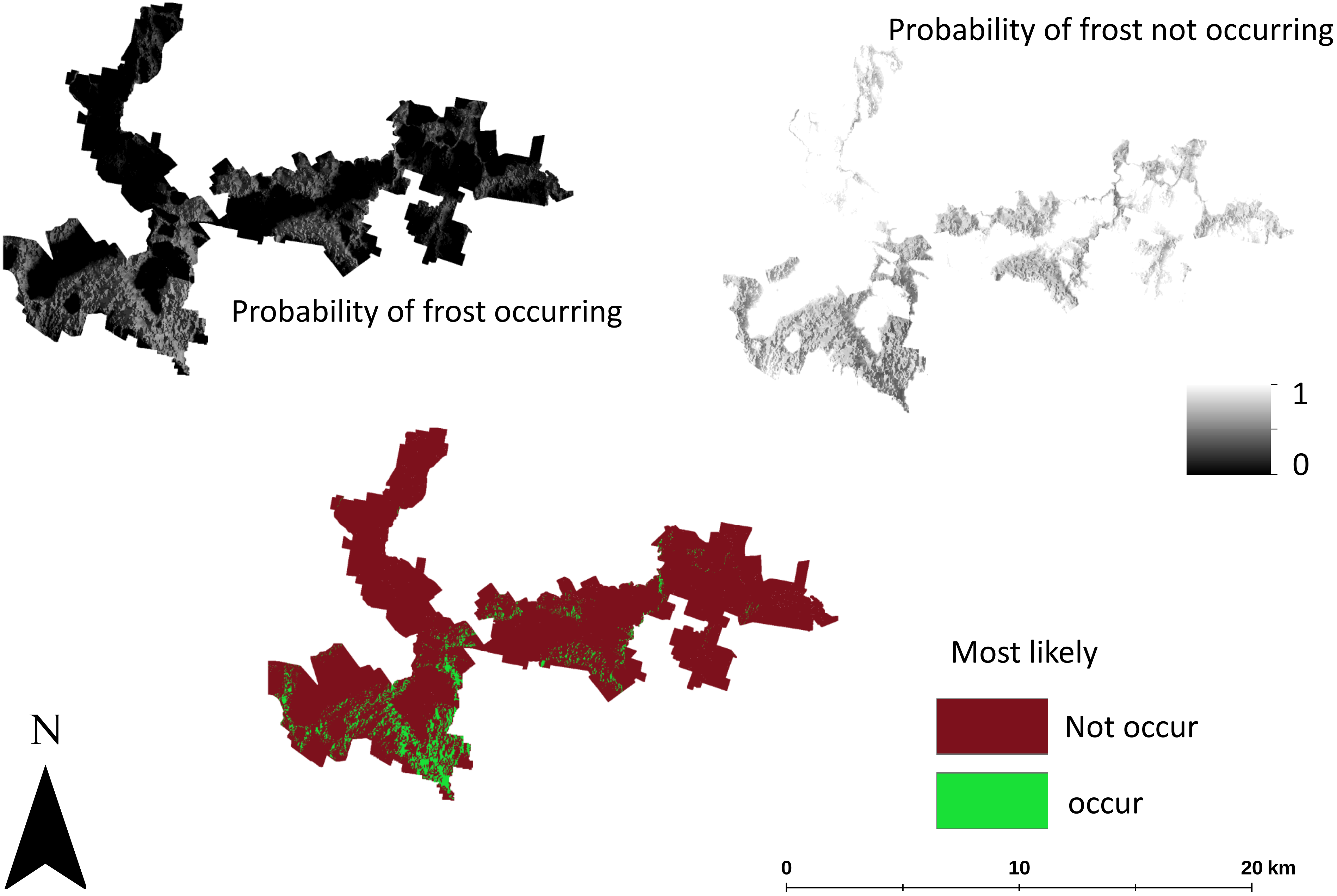

Land suitability assessment inputs for Hazelnuts: Meander Valley, Tasmania

Incidence of frost occurrence. Likelihood of not occurring (%)

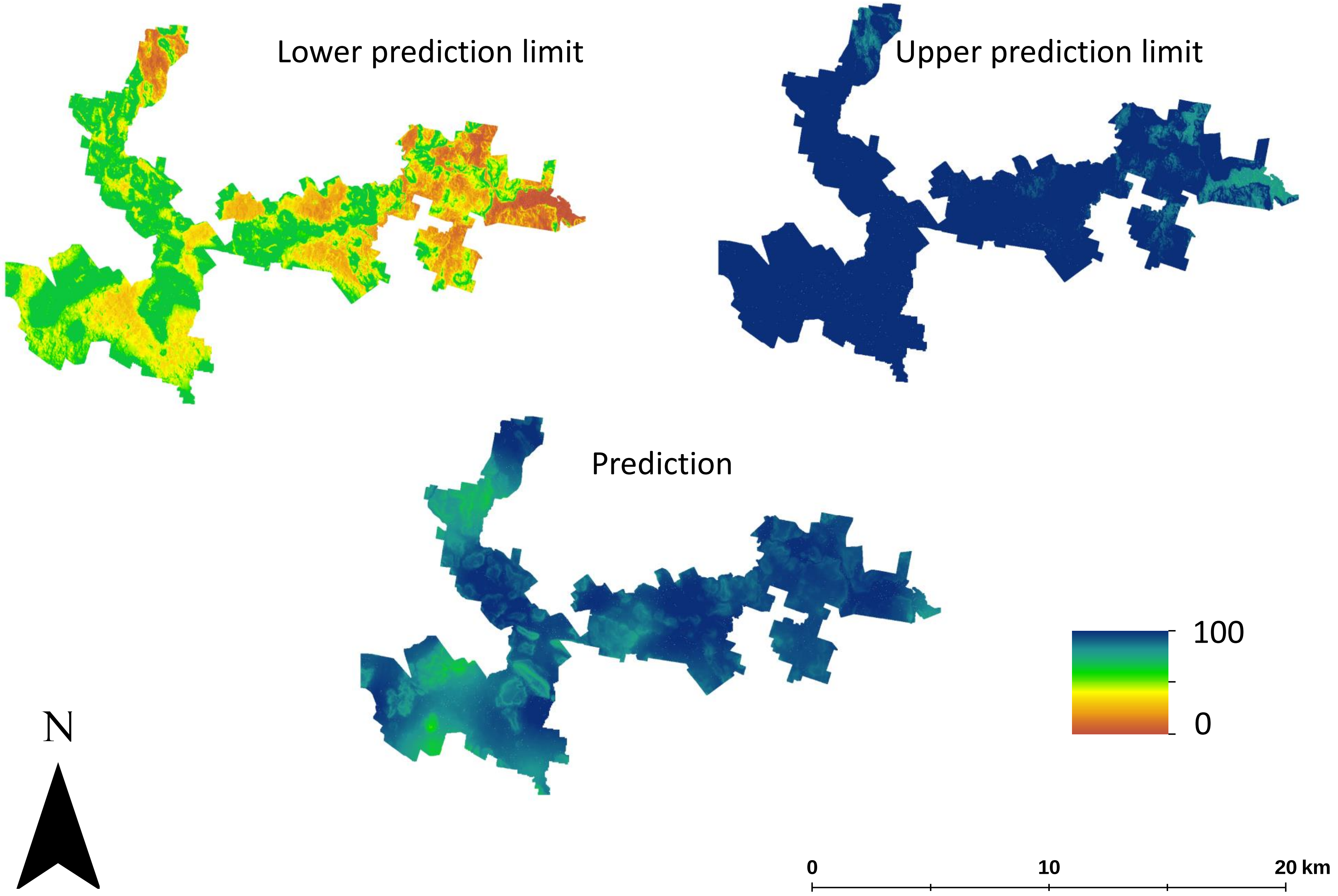

Land suitability assessment inputs for Hazelnuts: Meander Valley, Tasmania

Temperature (mean max monthly temp January, February)

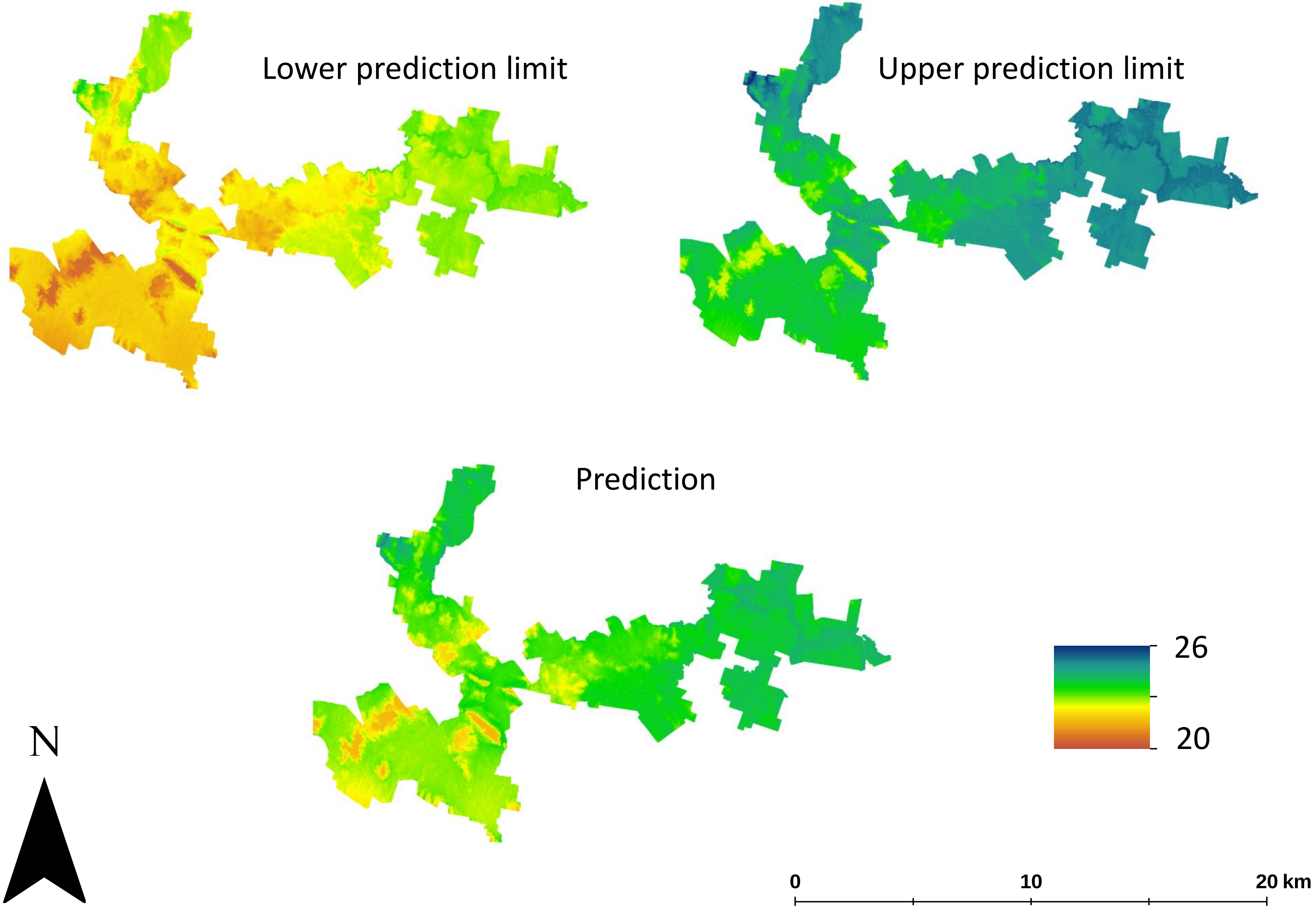

Land suitability assessment inputs for Hazelnuts: Meander Valley, Tasmania

Rainfall (mean rainfall for March)

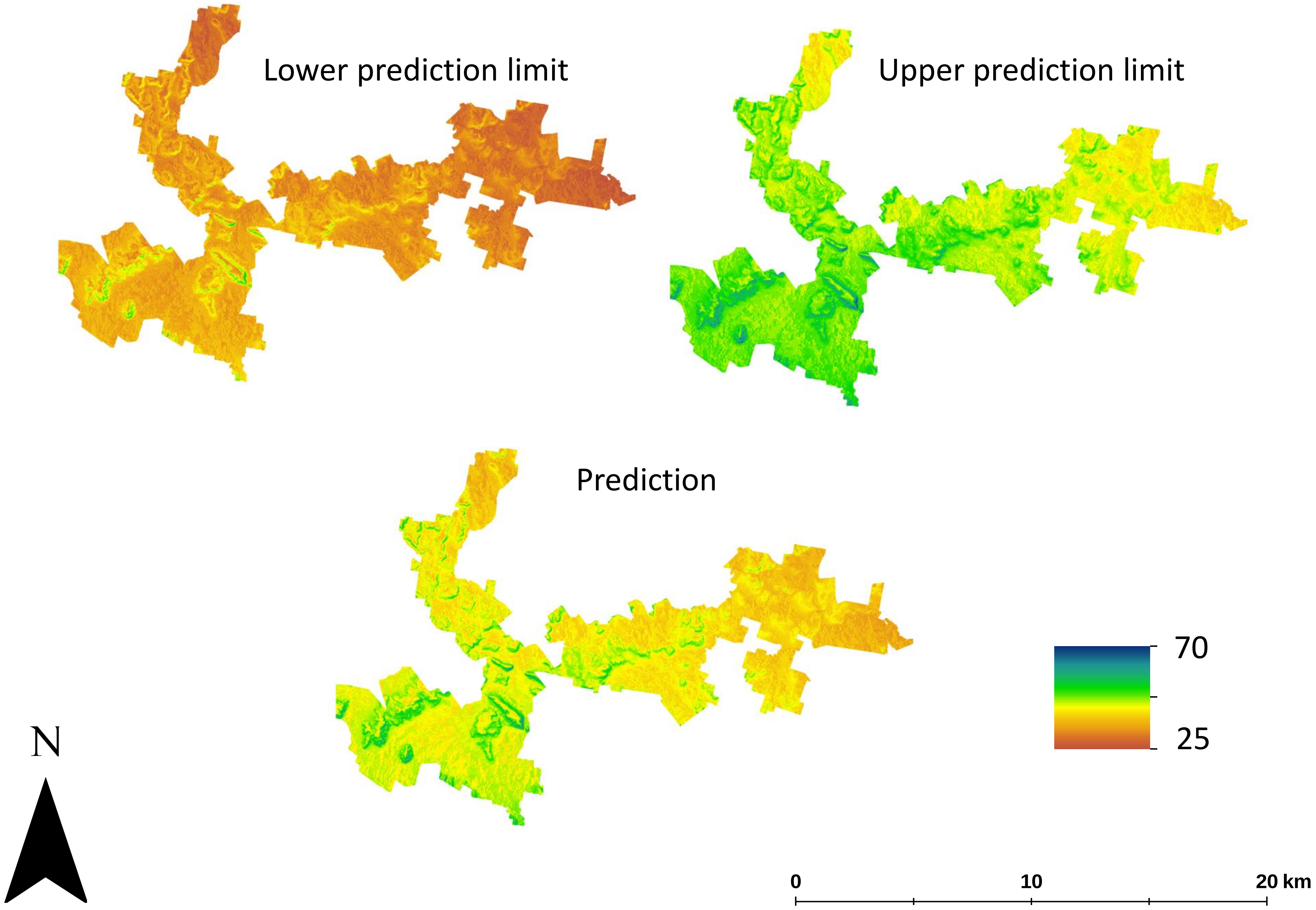

Land suitability assessment inputs for Hazelnuts: Meander Valley, Tasmania

Chill hours (April-August inclusive)

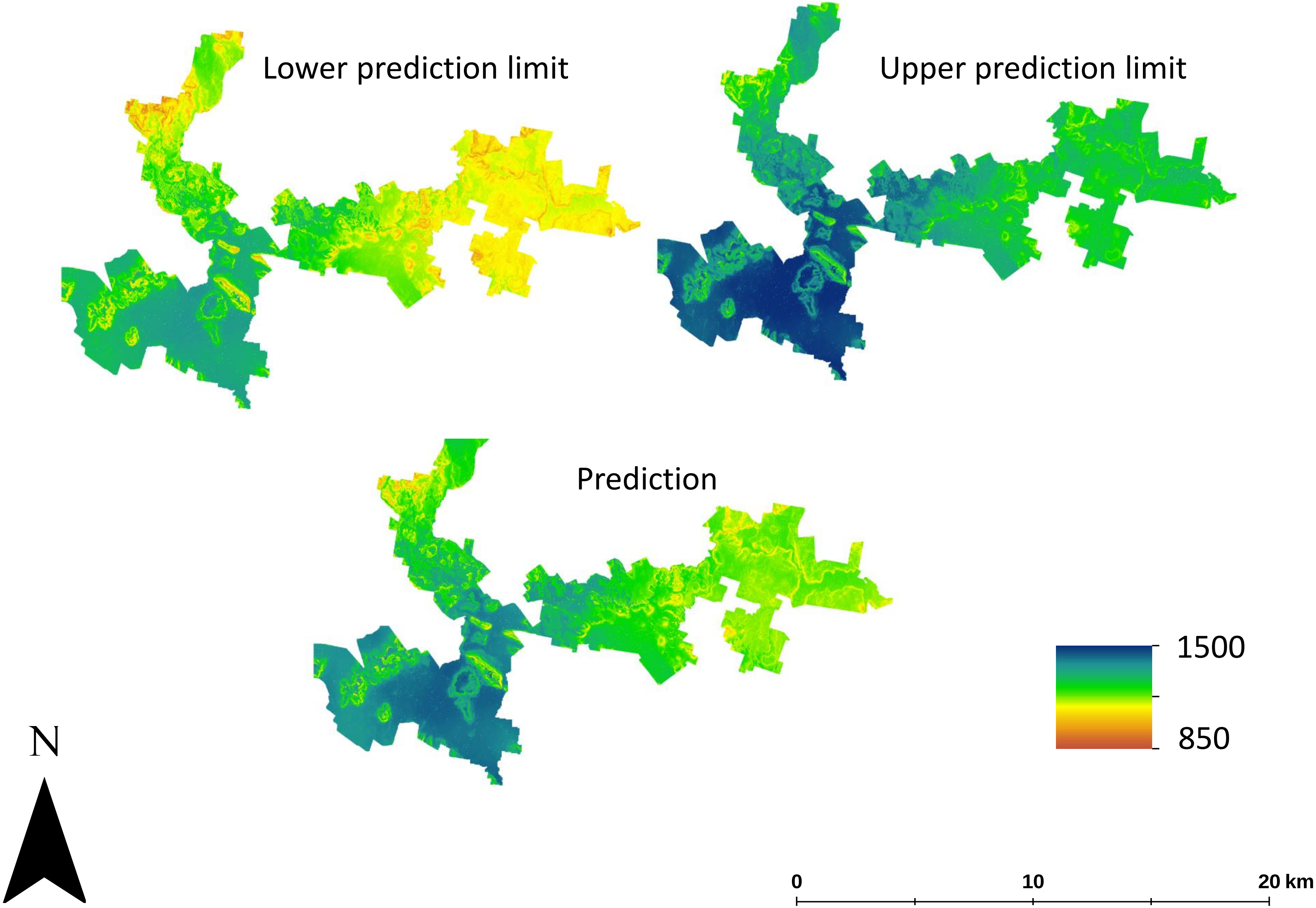

Supplement: Supplemental Information 1 [file peerj-03-1366-s001.pdf]
