# Supplementary material for: Taking account of uncertainties in digital land suitability assessment
Source: PeerJ. 2015 Oct 27;3:e1366. doi: 10.7717/peerj.1366 (PMC4627905; doi:10.7717/peerj.1366)

Most limiting factor occurrence for LSA: Hazelnuts. Meander Valley
Soil properties

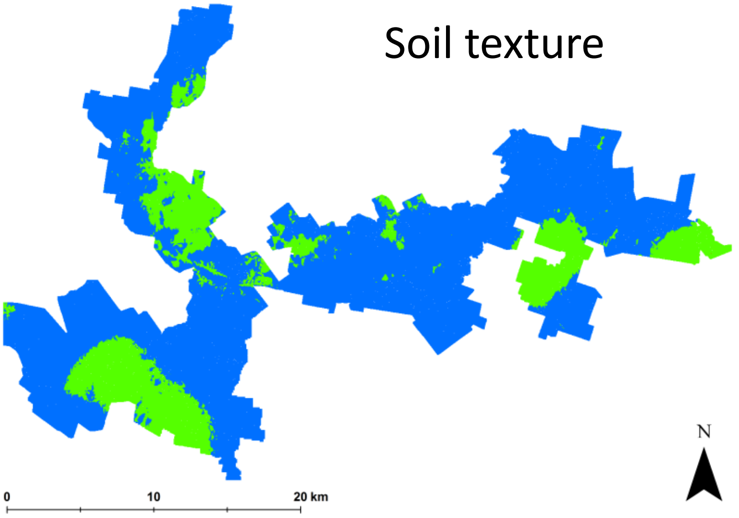

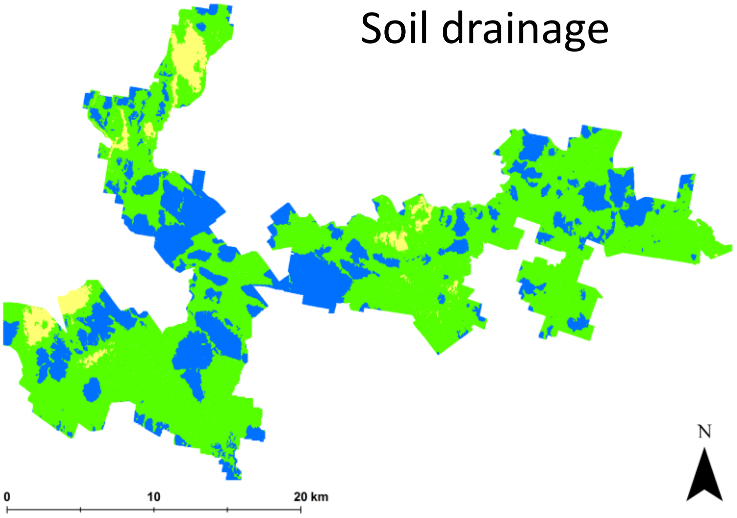

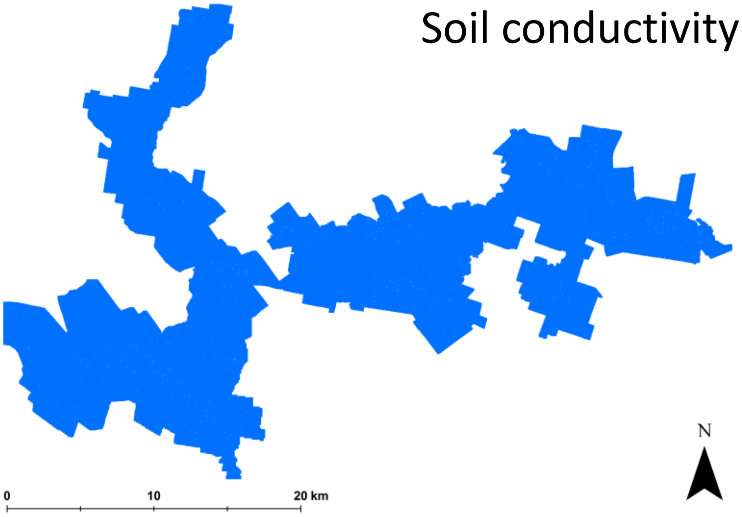

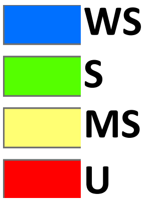

Most limiting factor occurrence for LSA: Hazelnuts. Meander Valley
Soil properties

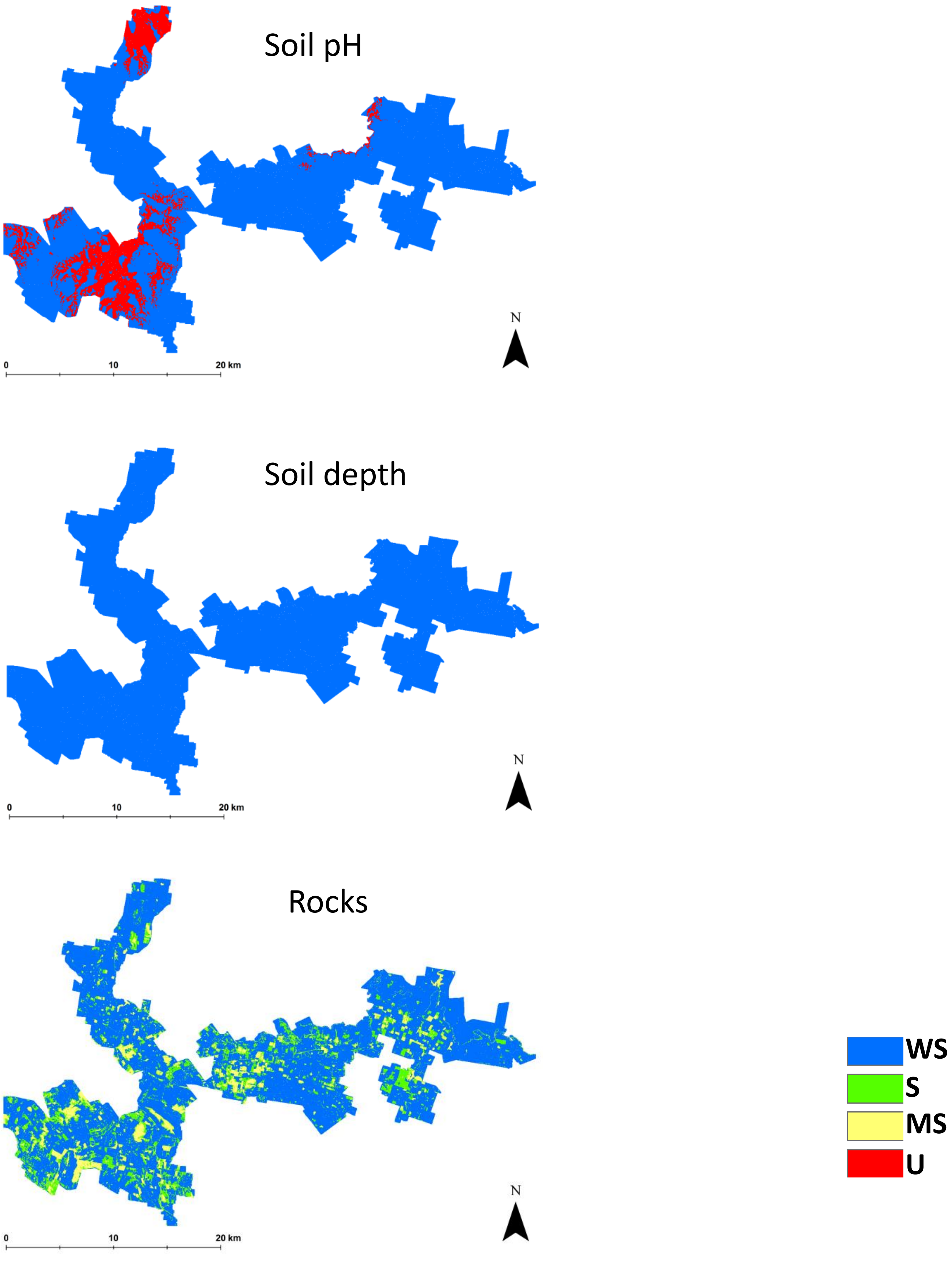

Most limiting factor occurrence for LSA: Hazelnuts. Meander Valley
Climate variables

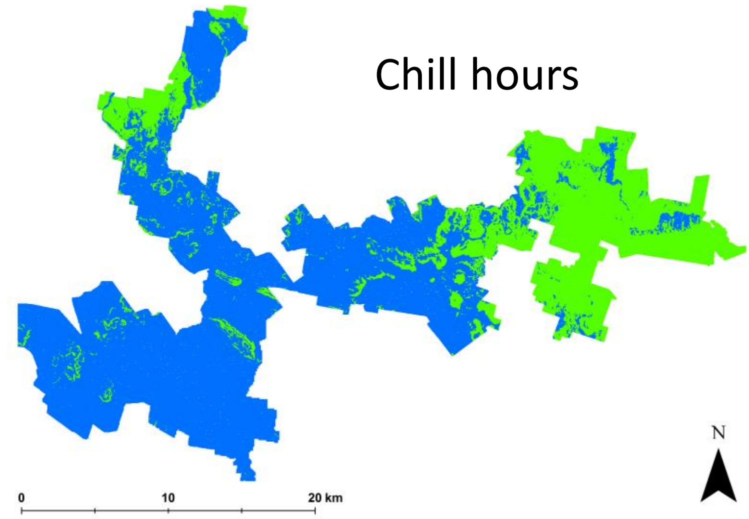

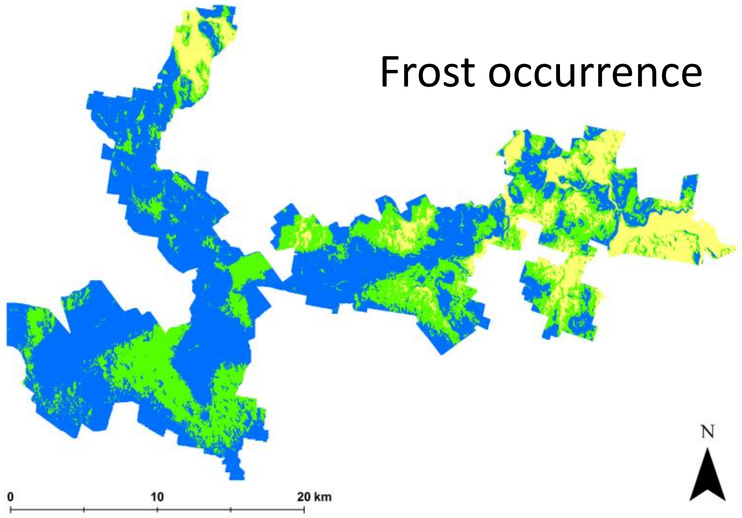

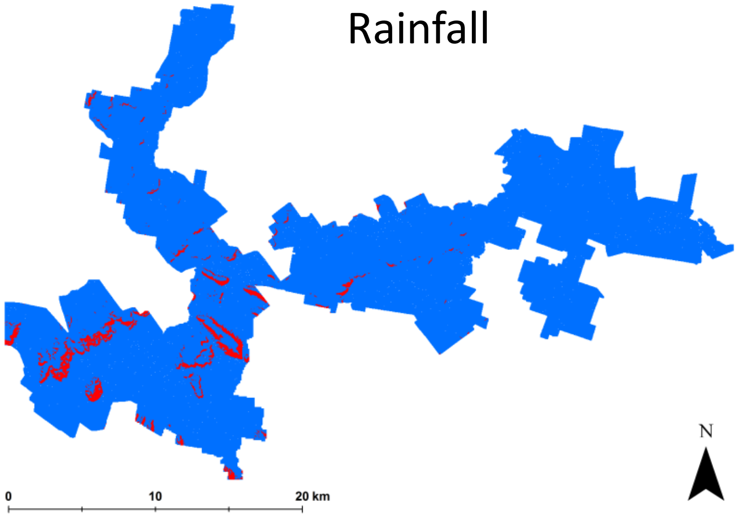

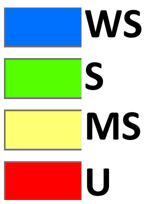

Most limiting factor occurrence for LSA: Hazelnuts. Meander Valley
Climate variables

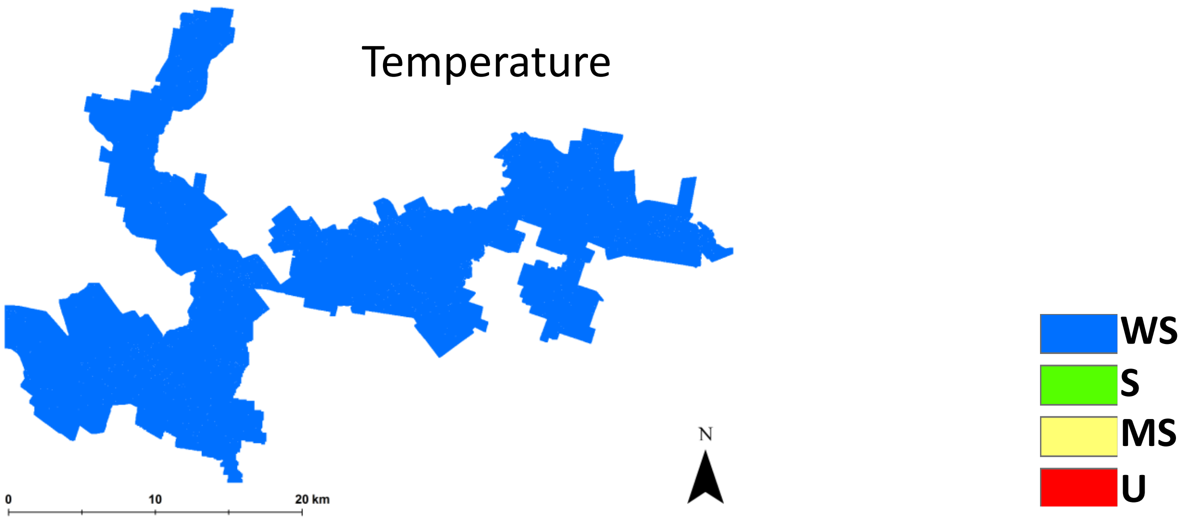

Supplement: Supplemental Information 2 [file peerj-03-1366-s002.pdf]
